# *FEBS Open Bio*: past, present and future

**DOI:** 10.1002/2211-5463.13326

**Published:** 2021-12-01

**Authors:** Duncan E. Wright, Félix M. Goñi, Mary Purton, László Fésüs, Johannes Buchner, John Mowbray, Miguel A. De la Rosa

**Affiliations:** ^1^ FEBS Open Bio Editorial Office Cambridge UK; ^2^ Department of Biochemistry Instituto BIOFISIKA (CSIC, UPV/EHU) University of the Basque Country Leioa Spain; ^3^ FEBS Press Cambridge UK; ^4^ Department of Biochemistry and Molecular Biology University of Debrecen Hungary; ^5^ Department of Chemistry Center for Protein Assemblies Technical University Munich Garching Germany; ^6^ Emeritus member of the Division of Biosciences University College London UK; ^7^ Institute for Chemical Research (IIQ) Scientific Research Centre Isla de la Cartuja (cicCartuja) Universidad de Sevilla‐CSIC Spain

## Abstract

In celebration of the 10th anniversary of FEBS Open Bio, we spoke to some of the key figures of the journal’s genesis, development, and its future direction, and recount here their thoughts and experiences. Prof. Félix. Goñi discusses the role of the FEBS Publication Committee in the journal's beginnings, Dr Mary Purton relates her experiences as the journal's Executive Editor, Prof. László Fésüs explains how the journal developed during his tenure as Chair of the Publication Committee, and Prof. Johannes Buchner looks forward to the future of FEBS Press and academic publishing. Finally, Prof. John (Iain) Mowbray describes his “Friday afternoon thought” to start a new journal.

## Graphical abstract

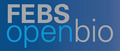

## Professor Félix M. Goñi

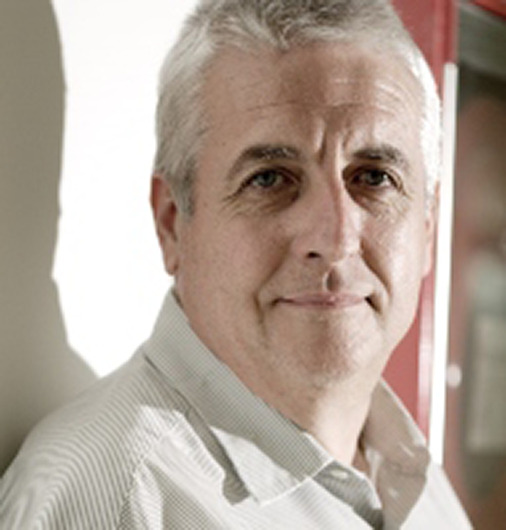

Professor Félix M. Goñi was Chair of the FEBS Publication Committee from 2006 to 2011 and played a central role in the discussions surrounding the approval for the new journal. Here, Professor Goñi shares with us the Publication Committee’s initial thoughts on starting a new, fully open access journal:

1. You were the Chair of the FEBS Publications Committee when *FEBS Open Bio* was conceived. What was the initial reaction of the Publications Committee to the suggestion that FEBS start a new open access journal?

At the time, several publishers were developing the concept of ‘cascade journals’, to facilitate transfer of manuscripts to more appropriate journals of the same publisher. Then, Iain Mowbray came one day in 2010 with what he called ‘a Friday afternoon thought’, consisting of starting a journal that would operate in cascade with *The FEBS Journal*, *FEBS Letters,* and *Molecular Oncology*, and provide a space for quality manuscripts that were not considered to require urgent publication (see below). The idea came with a name, ‘*FEBS Open Bio*’, indicating the great novelty, at the time, of an entirely Open Access journal, published in electronic version only. The Publications Committee response was unanimously positive, partly because Elsevier, then publisher of *FEBS Letters*, had also given its support. The then FEBS Secretary‐General, Professor Israel Pecht, had also stated his interest in the idea.

1. Many other open access journals were emerging at the same time as *FEBS Open Bio*. What was new in *FEBS Open Bio* compared with the others?

*FEBS Open Bio* was actually among the first entirely OA‐journals in Biochemistry and Molecular Biology. It also had the advantage of being linked to such distinguished journals as *The FEBS Journal*, *FEBS Letters* and *Molecular Oncology*, and sharing the same team of highly qualified reviewers.

1. And what was new compared with the other FEBS Press journals, namely *FEBS Letters*, *The FEBS Journal* and *Molecular Oncology*?

Open access, and faster publication (electronic only).

1. I hear that *FEBS Open Bio* had an extraordinary quick genesis, from original concept to its first issue. What was the driving force behind this accelerated process?

Iain Mowbray!

1. Did you have any reservations about starting *FEBS Open Bio*?

Frankly, we couldn’t know, at the time, what the future would bring to the whole publication business, in particular with the then revolutionary novelties of OA and electronic publishing. But we understood as well that we couldn’t stay away from what could (and indeed has) become the mainstream in academic publishing.

1. Did you anticipate the journal’s dramatic growth, in terms of both submissions and published articles?

Perhaps after a few drinks.

## Dr. Mary Purton

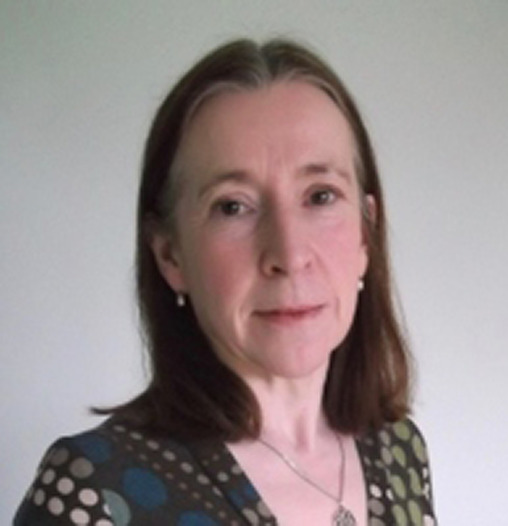

Dr Mary Purton was the journal’s Executive Editor from its beginnings in 2011 to the end of 2019. She is now FEBS Press Publisher, overseeing the performance of not only *FEBS Open Bio*, but all four FEBS Press journals. Dr Purton recalls the experience of successfully building and managing a new journal:

1. What were the main challenges in starting a new open access journal?

The main challenge when launching any new journal is to encourage authors to submit their research papers. Often journals have to invite review content to fill the first issues, before any original submissions are received.

This was not the case with *FEBS Open Bio* as authors who had submitted articles to the other FEBS journals (*The FEBS Journal*, *FEBS Letters* and *Molecular Oncology*) were, if rejected, invited to transfer their articles. The original editorial board of *FEBS Open Bio* comprised members of the editorial boards of the three other FEBS journals, and so authors were reassured that their papers would be in the hands of trusted editors. Those transferring after peer review were guaranteed a quick decision without further peer review. Within a few months of announcing the launch of *FEBS Open Bio*, there was a slow but steady stream of transferred manuscripts. Direct submissions were also welcome, and the first of these was submitted four months after the launch issue was published in December 2011.

Another challenge was to convince the committees responsible for indexing journals at ISI (Web of Science) and the National Library of Medicine that a journal publishing sound science was worthy of inclusion. At the time, many open access journals were viewed as ‘predatory’ and so the threshold for inclusion was set very high. The journal was accepted into Web of Science in 2014 but only gained full inclusion in Medline in early 2019.

1. What did you enjoy most while managing and editing *FEBS Open Bio*?

Starting a new journal, and a new type of journal, was a challenge but a very enjoyable one. There were no precedents, and so we had to find ways to make the transfer process work smoothly for all authors, even though *The FEBS Journal* was on another publisher’s platform. The editorial board were all committed to the aim of publishing sound papers rather than those with perceived ‘impact’, but this was new territory for us all.

1. Are there any individual success stories for the journal you’d like to share?

Journal Impact Factors are still used as a proxy of the quality of individual papers, even though the metric was not designed for this purpose. We published some highly cited articles in the years before *FEBS Open Bio* was first awarded an Impact Factor (June 2015). Some of these were transferred from other FEBS journals, and my colleagues on these journals still regret that they turned down these ‘citation classics’. I’m very grateful to the authors of these papers who trusted some of their best research to a journal without an Impact Factor. Other authors were delighted to see the interest their work received when they published open access for the first time.

1. How would you describe your experience in managing *FEBS Open Bio* for so many years? And what did you learn?

I thoroughly enjoyed my time with *FEBS Open Bio*. The journal was very much an experiment in the early days. However, it grew from publishing just 4 articles in the first volume, to 188 in volume 9, my last year as Executive Editor. Open access was still in its infancy in 2011, and I’ve been privileged to see it gaining acceptance and well on its way to become the standard way to publish. The cost of publication is still an issue for some authors, but with new business models for covering publication costs emerging, I hope that all scientific research will soon be open access.

## Prof. László Fésüs

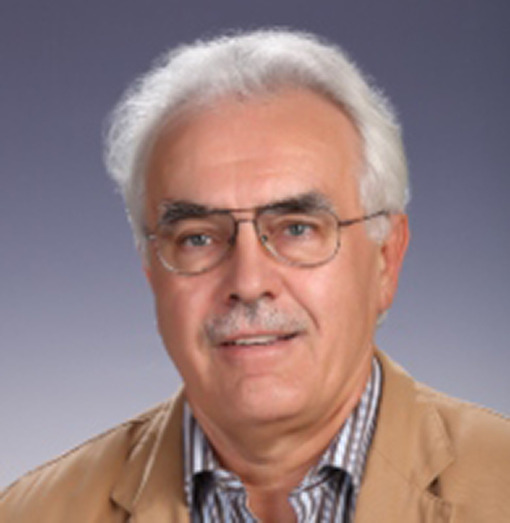

Professor László Fésüs was Chair of the Publications Committee from 2012 to 2019, and was thus well positioned to oversee the tremendous growth of *FEBS Open Bio*. Here, Prof. Fésüs shares his opinions on the success of the journal and the future of scientific publishing:

1. Why another FEBS journal? Was there a niche not covered by *The FEBS Journal*, *FEBS Letters* or *Molecular Oncology*?

At the time of launching *FEBS Open Bio* the niche appeared as an opportunity to publish, in a new open access journal, those scientifically sound manuscripts, which were originally submitted to *The FEBS Journal*, *FEBS Letters* or *Molecular Oncology* but were not published there because of limitations in topic, scope or novelty. FEBS was also motivated by the appearance and strengthening of the open access movement among scientists and in science publishing.

1. In just under ten years, *FEBS Open Bio* has published over 1200 manuscripts; did you anticipate that the journal would be so successful?

Personally, I expected continuous growth in the number of submitted and published articles. The transfer and submission dynamics during the first 3‐4 years predicted that *FEBS Open Bio* will publish more than 200 research articles by its 10^th^ anniversary. It was not anticipated that this number would be close to 300, as we have seen.

1. What do you think is the major achievement of *FEBS Open Bio*?

*FEBS Open Bio* has become a success story based on three major elements: the first is the FEBS brand, which was emphasized by joining of editors from *The FEBS Journal*, *FEBS Letters* and *Molecular Oncology* to its editorial board at the start; secondly, it was launched just in time before the mushrooming of open access journals began; and thirdly, the high professional skills of Mary Purton in managing the journal as Executive Editor.

Regarding its major achievements, I find it particularly important that direct submissions have gradually become the dominant source of published articles and the number of average citations to *FEBS Open Bio* research articles is close to those observed for the traditional FEBS journals.

1. How has the publishing landscape changed since *FEBS Open Bio* was launched?

There were predictions ten years ago that all subscription journals will flip to full open access and journal subscriptions will disappear within a decade. This did not happen and it seems the two interoperating journal landscapes will stay with us in the foreseeable future. Regarding the open access segment, many open access journals have been launched by both society and profit‐oriented publishers and new business models have appeared setting up complex networks of field‐specific journals (e.g. by MDPI, Frontiers), while new interdisciplinary mega journals with broad scope have been recently launched (e.g. Science Advances by AAAS, iScience by Cell Press, Natural Sciences by Wiley). FEBS Press journals face fierce competition in attracting quality manuscripts.

1. The FEBS website reads: ‘the [FEBS] journals have not only provided scientists with effective routes for research dissemination and assimilation, but also an income stream to fund the other programmes of FEBS’. Which reason was the major consideration in launching *FEBS Open Bio*?

The main reason for launching *FEBS Open Bio* was to provide a fast open access publishing route for researchers in the molecular life sciences. For several years, FEBS spent more on the journal than its generated income. It was expected that at some point revenues from *FEBS Open Bio* will be a sizable addition to the FEBS budget; the journal has reached this point before its 10^th^ anniversary.

1. How do you think academic publishing will change in the future?

In the Open Science era, much more emphasis will be on high standards in science publishing, requiring, among other things, full data and method transparency, appropriate citations of data and material, preregistration of studies, encouragement of replicative works, and introduction of open review processes. The traditional format of research articles may also change and publishing of raw data will be more common.

1. How would you describe your experience in chairing the FEBS Publications Committee for so many years?

My tenure as chair of the FEBS Publications Committee was exciting, challenging and rewarding. The committee managed to join the open access publishing trend by launching *FEBS Open Bio* and flipping *Molecular Oncology* to an open access journal, made strategic responses to Plan S developments, successfully negotiated a long‐term publishing contract providing financial stability to FEBS, established the FEBS Press platform for the FEBS journals and the position of the FEBS Press publisher, and launched the FEBS Network platform. We were fortunate to have the opportunity to contribute to the 50^th^ anniversary celebrations of FEBS, then of *The FEBS Journal* and of *FEBS Letters*, and to appoint new Editors‐in‐Chief to the FEBS Press journals while honouring their long‐serving predecessors.

## Professor Johannes Buchner

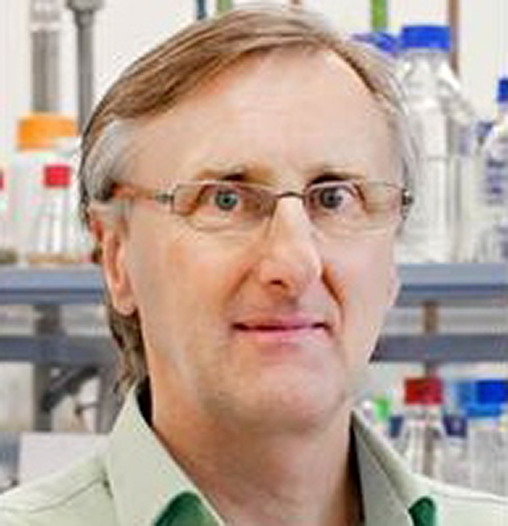

Professor Johannes Buchner has been the Chair of the FEBS Publication Committee since 2020. Here, he looks towards the future of *FEBS Open Bio*, FEBS Press and academic publishing:

1. What is the major driving force for the future development of *FEBS Open Bio*?

Providing a high‐quality outlet for the publication of scientific findings in the broad area of the molecular life sciences is the main driving force behind all four FEBS Press journals. We will try to maintain the strong reputation of the journals and at the same time try to develop the journals to meet current and future demands of scientists in Europe and around the world. For *FEBS Open Bio*, I envision continued growth and increase in impact as a premier open access journal.

1. How do you think academic publishing will change in the future?

Academic publishing will be of increasing importance to guarantee a publication process run by scientists for scientists where the most important aspect is the quality of the science. The independence from the big publishers allows us to develop the journal according to the needs of scientists without having to consider company interests.

1. How do you foresee the future development of *FEBS Open Bio* compared with its competitors?

Based on the strong upward trend in the past years, I envision that *FEBS Open Bio* will continue to grow in terms of publications and also impact. The professional editorial process with fast turnaround times and the reasonable publication fees are important aspects to support its development.

1. Do you consider open access to be superior to the traditional subscription model?

At the moment, both models have their values. We are living in a transition period and open access will be the prevailing model in the future. Certainly, as the name implies, the free access of scientific results to everybody is a great benefit. Paywalls then no longer prevent the dissemination of results and every voice can be heard. However, the financial burden is shifted to the authors and this is certainly a downside of the open access model.

1. What are the greatest challenges facing society journals today?

Society journals have been embedded in the long tradition of the respective societies. This has contributed to their reputation and the trust in the careful and fair handling of manuscripts. In the rapidly proliferating market of scientific journals where publishers create an increasing number of sister or baby journals to cover not only topics but also the range of impact factors, the value of society journals seems to be sometimes underestimated. It is our task to provide the right perspective and advertise the special character and benefits of our journals.

1. And what are your greatest challenges chairing the FEBS Publications Committee?

My position provides the link between the society and the editors. The challenge is to give the editors the highest amount of freedom and support and at the same time making sure that all journals develop along the same principles. My experience so far is an absolutely positive one. It is great to work with our editors, the editorial teams and the publication committee.

## Professor John (Iain) Mowbray

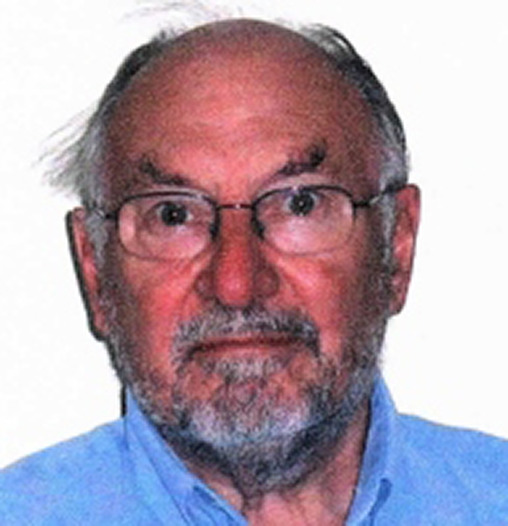

In 2010, Professor Mowbray, at that time Treasurer of FEBS, suggested that the society start a new fully open access journal, which became *FEBS Open Bio*. Here, he recounts the story behind the journal’s beginnings:

The Open Access (OA) publishing movement was espoused by Nobel Laureate Harold Varma and colleagues in the US when they established the Public Library of Science (PLoS) in 2001. This resulted in the founding of PLOS One in 2006 which by 2009 was getting 6000 submissions per year and publishing around 3500 papers. By 2010, it had become the largest scholarly journal in the world publishing around 700 manuscripts a month. There was manifestly no lack of enthusiasm for this competitor to traditional journal publishing. Since the money that FEBS used to fund its programmes such as Fellowships and Advanced Courses comes from the sales of our journals, it was obvious that this Web‐based publishing posed a threat to our income. Moreover, our managing editors confirmed that most of the citations to *The FEBS Journal* and *FEBS Letters* were from the United States. Richard Perham had also heard that the EMBO Director, Maria Leptin, was exploring an open access option for EMBO J and he reported that several distinguished scientists had refused to join *The FEBS Journal* editorial board because of our nonadherence to OA. It had become clear that we would have to embrace this OA development and I started discussions with Wiley‐Blackwell and Elsevier about the feasibility of doing this, since some of the criticism levied at PLOS One was that there was a significantly lower proportion of really novel contributions than in traditional high‐class journals (albeit they were sound enough). Thus, I formulated a proposal to the Publications Committee in February 2011 as follows:‐

### *FEBS OPENBIO* or *BIO‐OPEN*

An online open access journal publishing articles in molecular and cellular biology and cancer, approved by the FEBS family of Journals.

#### Instructions to authors

Articles for publication should be submitted in the normal way to The FEBS Journal, FEBS Letters and Molecular Oncology. Submissions judged by peer review to be sound and a contribution to knowledge but which do not meet the current perception of novelty required by these journals may be rapidly published online and made freely available to the scientific community in FEBS OPENBIO on payment of a publication charge.

This was supported by all members of the Publication Committee and it was considered that it would not compete with submissions to our other journals and so reduce their viability. Moreover, at the outset we would be using the current journal editors and so the editorial costs would be minimal. There were no takers for the name BIO‐OPEN and two votes for FEBS OpenBio (now FEBS Open Bio). To feed manuscripts until the journal could be self‐sufficient, we needed the agreement of the members of the editorial boards, which the managing editors of these journals duly sought and received.

I noted that PLOS One was charging $1400 for publication and after discussing this, settled on an initial article processing charge of €1200. Elsevier were much further ahead with their OA system, and since we needed to start quickly, I drafted a contract based on the one I had written for *FEBS Letters*; it was accepted by the 4th of July.

The other imperative was to set up an editorial office. The choice of Cambridge was easy because the building I’d hired for the *European Journal of Biochemistry*/*The FEBS Journal* office had several extra rooms which we’d never been able to sublet appropriately. Further, the presence there of experienced journal staff to offer collaborative support and the required communications facilities were present. The key was to find an experienced editorial manager to liaise between the journal editorial offices and the FEBS Open Bio publisher. By good fortune, Mary Purton, who’d previously worked as Editor for *Trends in Biochemical Sciences*, had just taken a temporary 3‐month position with *The FEBS Journal,* and I was able to offer her a permanent position with *FEBS Open Bio* from 1 December 2011.

The first paper was in proof on 23rd of October 2011.

## Author contribution

DEW and MAD wrote the questions and prepared the manuscript. The other authors answered the indicated questions.

